# Recombinant Ricin Toxin Binding Subunit B (RTB) Stimulates Production of TNF-α by Mouse Macrophages Through Activation of TLR4 Signaling Pathway

**DOI:** 10.3389/fphar.2020.526129

**Published:** 2020-09-08

**Authors:** Na Xu, Kaikai Yu, Haotian Yu, Jianxu Zhang, Yang Yang, Mingxin Dong, Yan Wang, Ying Chang, Yucheng Sun, Yanguang Hou, Chengbiao Sun, Jiayu Wan, Wensen Liu

**Affiliations:** ^1^ Institute of Military Veterinary Medicine, Academy of Military Medical Sciences, Zoonosis Prevention and Control Key Laboratory, Changchun, China; ^2^ Jilin Medical University, Jilin, China; ^3^ Institute of Translational Medicine, First Hospital of Jilin University, Changchun, China

**Keywords:** ricin toxin binding subunit B, macrophage, TLR4, cytokine, signaling pathway

## Abstract

Ricin toxin binding subunit B (RTB) is a galactose-binding lectin protein derived from the beans of the castor oil plant (*Ricinus communis*). Our previous studies have reported a direct immunomodulatory effect of recombinant RTB, which stimulates RAW264.7 cells to produce cytokines including TNF-α. However, the role of RTB in innate immune response and its specific mechanism have not been reported in detail. In this work, the results showed that RTB treatment of macrophages significantly increased TLR4 protein levels. RTB also activated TLR4 downstream events, including MyD88, IRAK, and TRAF6, resulting in macrophage activation and TNF-α production. This process is reflected in the increase of IκB phosphorylation. TLR4 knockdown macrophages treated with RTB exhibited greatly reduced IκB phosphorylation and TNF-α secretion. Moreover, treatment with MyD88 inhibitor also suppressed TNF-α production. The docking of RT and TLR4 was simulated by computer, and the contact residues were concentrated on RTB. Our results suggest that recombinant RTB can activate mouse macrophages to secrete TNF-α through activation of NF-κB *via* the TLR4 signaling pathways.

## Introduction

Ricin is a plant toxin extracted from castor bean (*Ricinus communis*), which belongs to type II ribosome-inactivating proteins. The ricin holotoxin consists of a toxic moiety A chain (RTA) and a lectin moiety B chain (RTB) linked *via* a disulfide bond. (Lord and Spooner, 2011). The A-chain has N-glycosidase enzymatic activity and causes protein synthesis arrest in mammalian cells. The B-chain is a galactose-binding lectin protein that binds to galactosyl moieties on the eukaryotic cell membrane (Stirpe and Barbieri, 1986; Lord et al., 2003). Our previous studies reported that recombinant RTB is an immunomodulatory stimulus that stimulates the RAW264.7 mouse macrophage cell line to produce inducible NOS (iNOS), tumor necrosis factor–α (TNF-α) and interleukin 6 (IL-6) *via* signaling pathways that may involve protein tyrosine kinase, NF-κB, and JAK-STAT activation (Liu et al., 2013; Xu et al., 2013). However, the role of RTB in innate immune response and its specific mechanism have not been understood very well.

Macrophages are important immune cells, which play an important role in anti-infection, anti-tumor and immune regulation as the first line of defense of innate immunity. Activated macrophages release many types of cell factors to remove pathogens (Akira et al., 2006; Mogensen, 2009). When pathogens invade the body, a series of intracellular signal transduction pathways are activated by various pattern recognition receptors (PRRs) expressed on macrophages. Toll-like receptor (TLR), a member of the PRR family which involved in stimulating innate immune response (Horng et al., 2002). When TLRs are activated, they require adaptor molecules, including myeloid differentiation primary response protein 88 (MyD88), TIR-domain-containing adaptor-inducing interferon-β (TRIF), IL-1R–associated kinases (IRAKs), and TNF receptor associated factor 6 (TRAF6) (Motshwene et al., 2009; Pålsson-McDermott and O’Neill, 2009; Lin et al., 2010). MyD88 is recruited by TLR4, and TNF-α is secreted following LPS mediates the activation of MyD88-dependent pathway by stimulating TLR4 (Park et al., 2010).

Since RTB has been shown to induce TNF-α production by RAW264.7 cells, we investigated whether TLR4 signaling pathway plays a key role in RTB-mediated TNF-a secretion.

## Materials and Methods

### Protein

RTB was cloned and expressed in Escherichia coli as described in our previous reaserch with a slight modification (5). The protein was purified using a nickel column (Ni-NTA; General Electric Corp., Boston, MA, US). Endotoxin was removed using Detoxi-Gel™ Endotoxin Removing Gel (Invitrogen, Frederic, MD, USA). The endotoxin contamination in the RTB was <0.03 pg/μg to exclude the possibility of LPS contamination.

### Cell Culturing

RAW264.7 cells were incubated in RPMI-1640 medium (Gibco, Carlsbad, CA, USA), containing 10% fetal calf serum (FCS) (Hyclone, Logan, UT, USA), 100 U/mL of penicillin, and 100 U/mL of streptomycin at 37°C in a humidified atmosphere containing 5% CO_2_ and 95% air.

### Cell Viability Assay

RAW264.7 cells were plated into 96-well plates at 5 × 10^4^ cells/well and cultured for 24 h. After addition of various concentrations of RTB (12.5, 25, 50, and 100 μg/mL), the cells were incubated with 10 μL of Alamar Blue cell viability reagent (Invitrogen, Frederic, MD, USA) in medium for 24 h at 37°C. The absorbance was measured at wavelengths of 570 and 610 nm.

### RNA Interference

RAW264.7 cells were transfected with psiRNA-mTLR4 to knockdown the TLR4 gene and with empty plasmid psiRNA-LucGL3 as control. Zeocin selection was used to collect the stably transfected RAW264.7 cells. The expression of TLR4 was examined by RT-PCR and western blotting.

### Western Blot Analysis

RAW264.7 cells were pretreated with or without RTB for the indicated time period, lysed with 100 µL of RIPA lysis buffer (Beyotime Institute of Biotechnology, Jiangsu, China) in the presence of protease inhibitor. Protein samples were separated by 10% SDS-PAGE and transferred to polyvinylidene difluoride (PVDF) membranes (Merck-Millipore, Darmstadt, Germany). The PVDF membranes were blocked with 5% non-fat milk (BD Biosciences, Franklin Lakes, NJ, USA) for 1 h at room temperature and then incubated with TLR4, MyD88, IRAK, TRAF6, or pIκB antibody (Cell Signaling Technology, Danvers, MA, USA) followed by appropriate secondary antibodies. Immunodetection was performed using ECL reagents (GE Healthcare).

### Cytokine Assays

To evaluation the TNF-a secretion induced by RTB, RAW264.7 cells or TLR4- siRNA treated RAW264.7 cells were plated into 24-well plates, stimulated with RTB (50 μg/mL), the MyD88 inhibitor Pepinh-MYD (25 mM) (InvivoGen, San Diego, CA, USA) or TAK-242 (50 nM) (MedChemExpress, Monmouth Junction, NJ, USA) for 24 h. Cytokine production in the supernatants was measured *via* ELISA, according to the manufacturers’ instructions (Biolegend, San Diego, CA, USA).

### Flow Cytometry

RAW264.7 cells were stimulated with or without RTB, and incubated with PE-conjugated anti-mouse TLR4 (Ebioscience, San Diego, CA, USA) for 30 min at 4°C. PE-labeled IgG antibody was used as control. Flow cytometry analysis was performed using a CytoFLEX (BECKMAN COULTER) flow cytometer.

### Computer Simulation of Molecular Docking

The amino acid sequences of TLR4 and RT were obtained from the NCBI database (https://www.ncbi.nlm.nih.gov). Protein modeling was performed using the Swiss-Model (swissmodel.expasy.org) online service *via* the TLR4 and RT amino acid sequences. An appropriate template was selected to model the 3D structure of the protein according to the sequence alignment. Then the protein structure was optimized by Discovery Studio 4.5 (Acclery, Inc.). The Discovery Studio Client was used to visualize the protein structure model, and the ZDOCK algorithm was used to simulate the intermolecular docking. The results of molecular docking were optimized and analyzed by using RDOCK.

### Statistical Analysis

One-way ANOVA followed by a *post hoc* comparison using the least significant difference (LSD) and independent t-test were performed for data comparison using SPSS 11.0 statistical software(IBM, Armonk, NY, USA). All graphical illustrations were generated in Graph Pad Prism 6. Significance was indicated as follows: *p ≤ 0.05; **p ≤ 0.01; ***p ≤ 0.001.

## Results

### Cell Viability Assay

**Figure 1:**
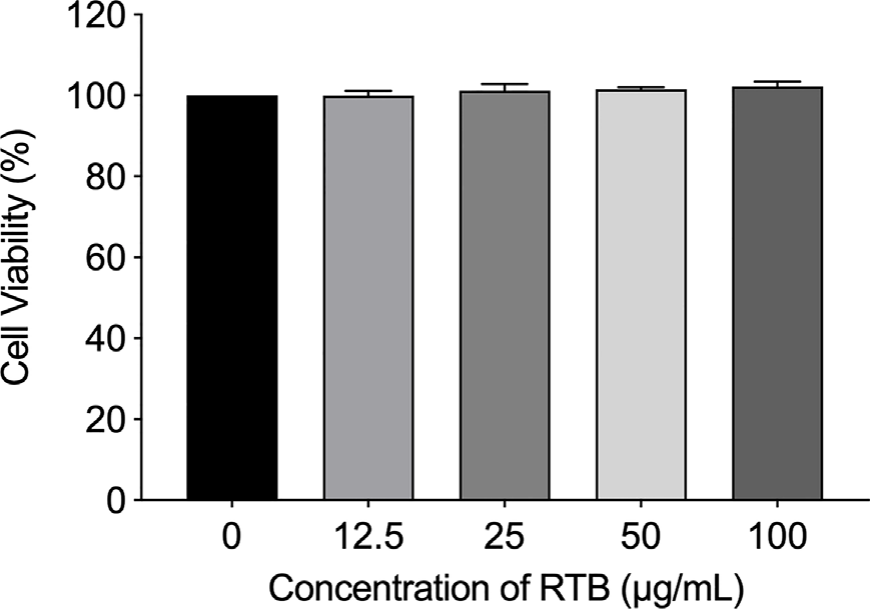
Effects of RTB on cell viability. Cells were treated with various concentrations of RTB for 24 h. Cell proliferation was analyzed by Alamar Blue assay. Cell viability without RTB treatment (control) was taken as 100%. Data represent the mean ± SD from three representative experiments.

Alamar Blue assay was used to measure cell viability and cytotoxicity. The results showed that RTB was not toxic to RAW264.7 cells at concentrations of 12.5−100 μg/mL (**Figure 1**). Based on this result, 50 μg/mL was used in subsequent experiments.

### The Effects of RTB on TLR4 Expression in RAW264.7 Cells

**Figure 2:**
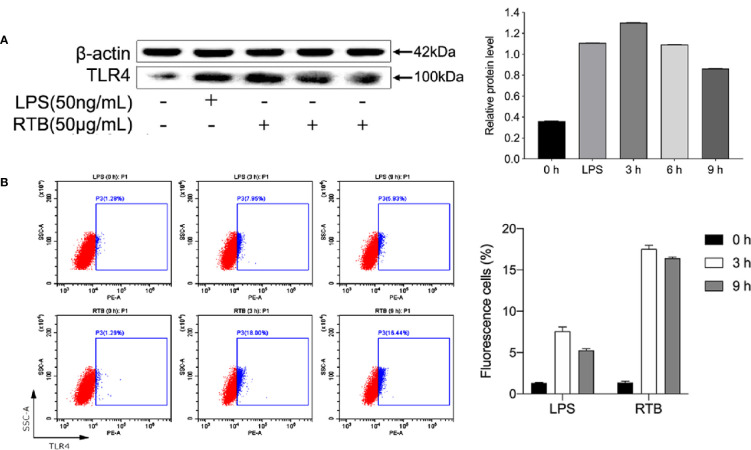
The analysis of TLR4 expression on RTB-treated RAW264.7 cells. **(A)** Expression levels of TLR4 were detected by western blotting. Cells were treated with RTB (50 μg/mL) for 3, 6, or 9 h or LPS (50 ng/mL) for 6 h. Whole cell lysates were prepared and TLR4 protein expression checked by western blotting using a specific antibody against TLR4 or β-actin. Relative protein level represents the relative ratio of expression of each protein versus actin. **(B)** TLR4 expression on RAW264.7 cells were analysed by flow cytometry. Cells were treated with RTB (50 μg/mL) for 3 or 9 h or LPS (50 ng/mL) for 3 h. TLR4 expressions were examined by flow cytometry. Representative results from one of three experiments with similar results are shown. Data represent the mean ± SD from three representative experiments.

To study whether TNF-α production by RAW264.7 cells induced by RTB is related to the TLR4 signaling pathway, we first determined the expression of TLR4. Enhanced protein expression induced by RTB was observed. The maximum expression of TLR4 was observed at 3 h and then decreased gradually up to 6 h (**Figure 2**).

### RTB Activates the TLR4 Signaling Pathway in RAW264.7 Cells

**Figure 3:**
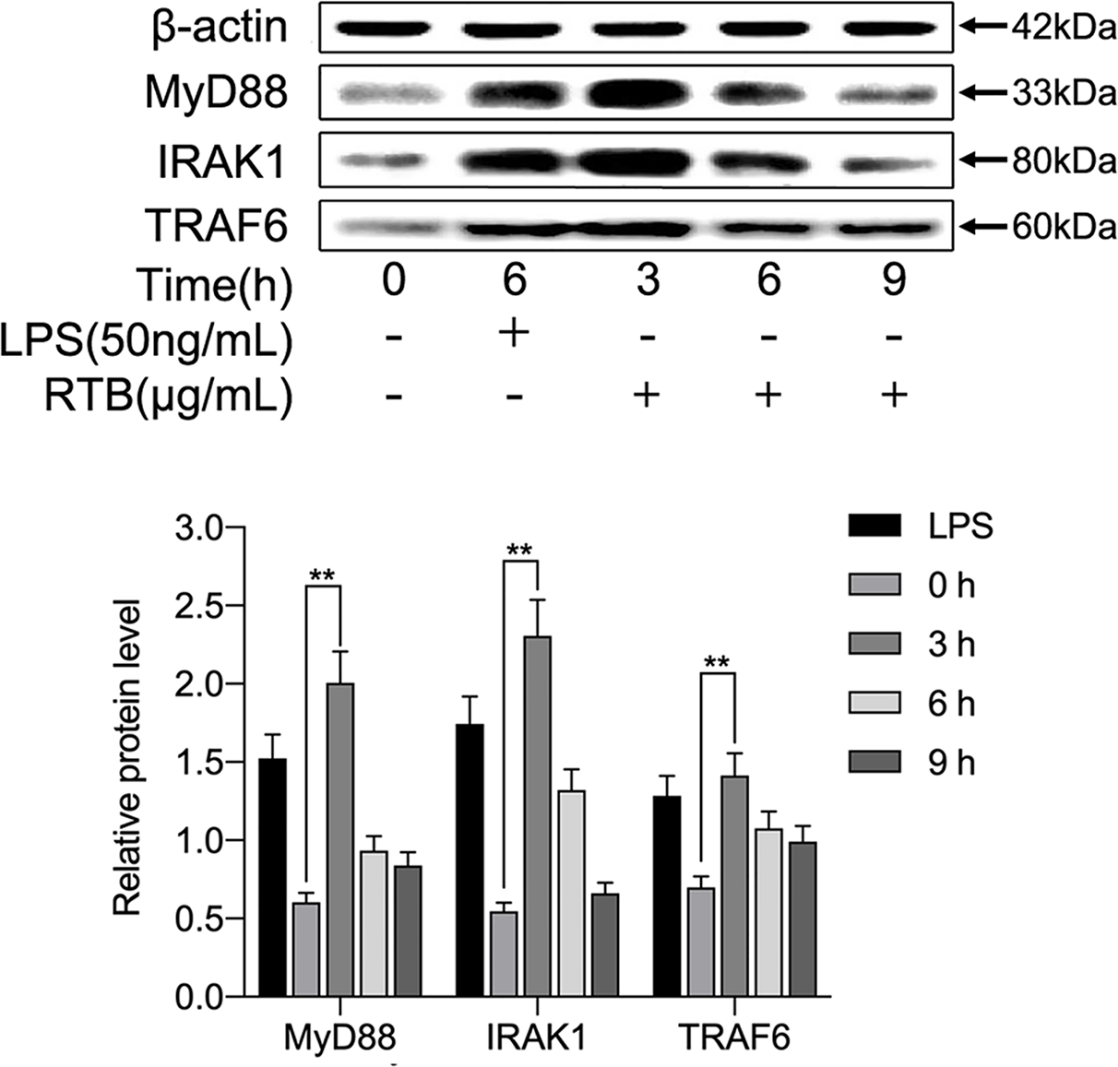
Effect of RTB on the expression of TLR4-signaling pathway components in RAW264.7 cells. RAW264.7 cells were treated with RTB (50 μg/mL) for 3, 6, or 9 h or LPS (50 ng/mL) for 6 h. Expression levels of MyD88, IRAK1, and TRAF6 were determined by western blotting. Relative protein level represents the relative expression of each protein versus actin. Representative results from one of three experiments with similar results are shown. Data represent the mean ± SD of three replicates. Significance was indicated as follows: p ≤ 0.01 (**).

To determine whether RTB activates the TLR4 signaling pathway, we examined whether downstream signaling molecules MyD88, IRAK, and TRAF6 were stimulated by RTB. As shown in **Figure 3**, RTB-treated macrophages showed increases in the expression of MyD88, IRAK, and TRAF6. MyD88, IRAK, and TRAF6 protein expression all showed a similar result to TLR4, reaching their maximum level at 3 h and then decreasing gradually up to 6 h.

### TLR4-Dependent TNF-α Production Following RTB *S*timulation

**Figure 4:**
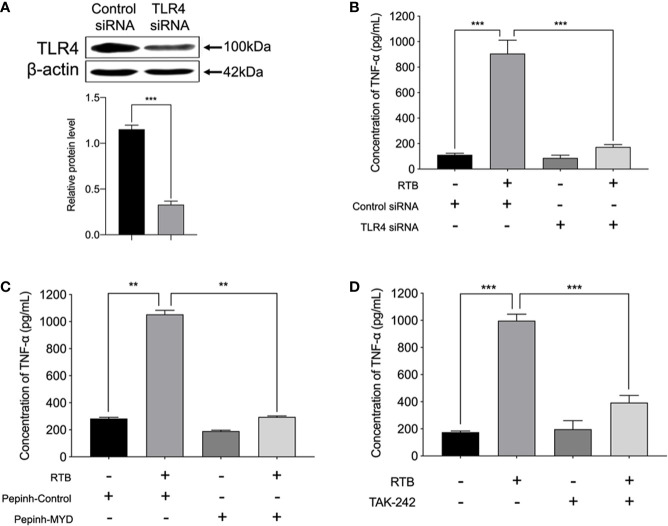
TNF-α production in Raw264.7 cells treated with RTB. **(A)** Cells were stably transfected with control siRNA or siRNA specific for TLR4.Western blotting analysis of TLR4 in cell lysates. **(B)** TNF-α production in stably-transfected RAW264.7 cells treated with RTB. TLR4 siRNA stably-transfected RAW264.7 cells were treated with RTB (50 μg/mL) for 24 h. TNF-α production was measured by ELISA. Data are presented as mean ± SD of three replicates. Shown are representative results from one of three experiments with similar results. **(C)** Raw264.7 cells were treated with or without the MyD88 inhibitor Pepinh-MYD (25 mM) and incubated for 24 h and then stimulated with RTB (50 μg/mL) for 24 h. TNF-α production was measured by ELISA. Data are presented as mean ± SD of three replicates. Representative results from one of three experiments with similar results are shown. **(D)** Raw264.7 cells were treated with or without another MyD88 inhibitor TAK-242 (50 nM) and incubated for 24 h and then stimulated with RTB (50 μg/mL) for 24 h. TNF-α production was measured by ELISA. Data are presented as mean ± SD of three replicates. Representative results from one of three experiments with similar results are shown. Significance was indicated as follows: **p ≤ 0.01; ***p ≤ 0.001.

We further determined whether TNF-α secretion was induced through RTB-activated TLR4 signaling pathways. We examined the effect of treatment with a TLR4 siRNA or MyD88 inhibitor on the RTB-activated induction of TNF-α in RAW264.7 cells. A TLR4 knockdown RAW264.7 cell line was created by stable transfection with psiRNA-mTLR4 (**Figure 4A**). TNF-α expression was significantly reduced in TLR4- siRNA or MyD88 inhibitor treated macrophages (**Figures 4B–D**).

### TLR4-Dependent Activation of NF-κB Following RTB Stimulation

**Figure 5:**
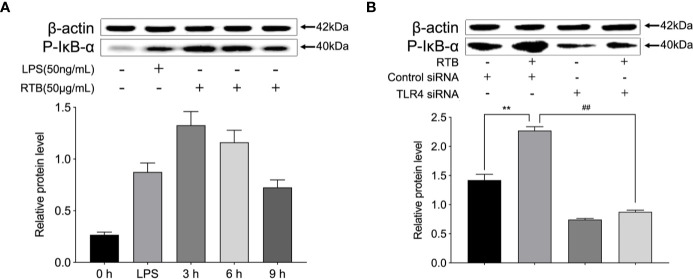
pIκB expression in Raw264.7 cells treated with RTB. **(A)** Raw264.7 cells were treated with RTB (50 μg/mL) for 3, 6, or 9 h or LPS (50 ng/mL) for 6 h. Levels of pIκB were determined by western blotting. The relative protein levels are expressed as the relative ratios versus actin. Representative results from one of three experiments with similar results are shown. Values are the mean ± SD of three replicates. **(B)** TLR4 stably transfected RAW264.7 cells were stimulated with RTB (50 μg/mL), and pIκB levels were determined by western blotting. Representative results from one of three experiments with similar results are shown. Values are the mean ± SD of three replicates. **p < 0.01 compared to control siRNA group. ^##^p < 0.01 compared to control siRNA treated with RTB.

To further explore whether TLR4 is involved in RTB-induced macrophage activation, IκB activation was measured to identify signaling changes. As shown in **Figure 5**, the expression of phosphorylated IκB was up-regulated within 3 h of RTB stimulation. In contrast, expression of phosphorylated IκB in TLR4- siRNA treated macrophages was substantially reduced.

### Computer Simulation of the Interface of RT and TLR4

**Figure 6:**
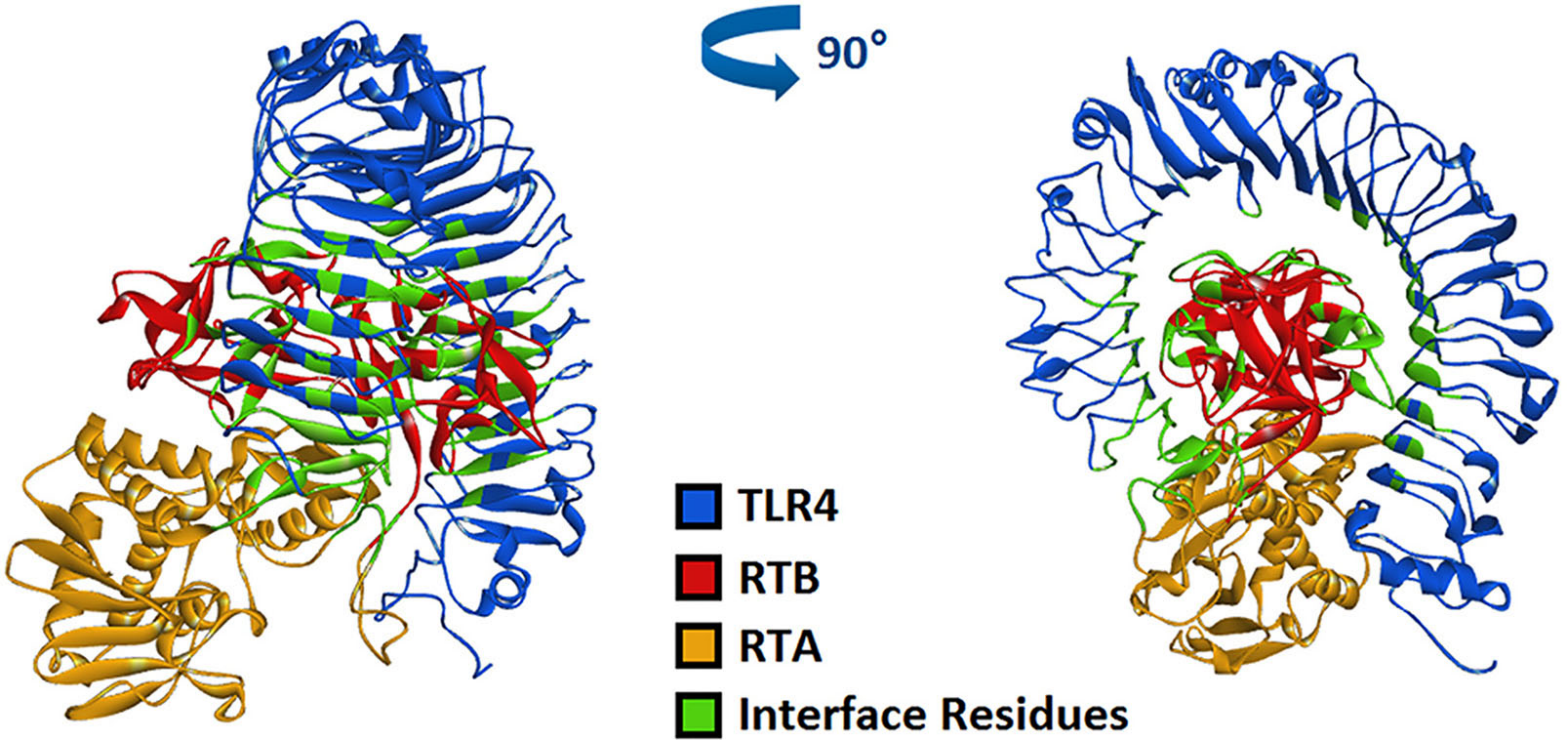
Computerized simulation to reveal the complex of TLR4 bound by RT. RT formed a contact interface with residues mainly in RTB domain.

Model and optimize the structural models of RT (ID: 2vlc) and TLR4 (ID: 3vq1) through the Swiss-Model online server. The Dock and Analyze Protein Complexes tool panel in Discovery Studio was used to dock proteins with the Dock Proteins (ZDOCK) protocol and display. Then, the ZRANK analysis tool was used to determine the complex structure of RT and TLR4, and the conformation of the complex was optimized by the RDOCK algorithm, and the optimal docking structure of RT and TLR4 was determined. The binding interface and area of RT and TLR4 are shown in **Figure 6** and **Table 1**.

**Table 1:** The binding interface and area of RT and TLR4.

From	To	RT Domain	Distance	Category
TLR4:LYS475:NZ	RT:ASP336:OD2	RTB	3.06226	Hydrogen bond; electrostatic
TLR4:LYS354:NZ	RT:ASP500:OD2	RTB	3.16169	Hydrogen bond; electrostatic
TLR4:ARG74:NH1	RT:GLU30:OE2	RTA	4.81973	Electrostatic
TLR4:ARG416:NH2	RT:GLU128:OE2	RTA	4.74054	Electrostatic
TLR4:ARG512:NH1	RT:GLU134:OE1	RTA	3.78613	Electrostatic
TLR4:LYS533:NZ	RT:ASP41:OD2	RTA	5.13394	Electrostatic
TLR4:LYS475:NZ	RT:TRP351	RTB	4.49247	Electrostatic
TLR4:TYR37:OH	RT:PRO297:O	RTB	3.38661	Hydrogen bond
TLR4:ARG86:NH1	RT:SER543:O	RTB	3.34691	Hydrogen bond
TLR4:SER104:OG	RT:SER418:OG	RTB	2.51362	Hydrogen bond
TLR4:ARG337:NE	RT:SER520:OG	RTB	3.36619	Hydrogen bond
TLR4:ARG337:NH1	RT:SER520:OG	RTB	3.32147	Hydrogen bond
TLR4:TYR454:OH	RT:LYS354:O	RTB	2.64150	Hydrogen bond
TLR4:LYS503:NZ	RT:LYS354:O	RTB	3.21736	Hydrogen bond
TLR4:SER545:OG	RT:ASN374:OD1	RTB	3.38933	Hydrogen bond
TLR4:SER300:N	RT:SER54:OG	RTA	3.17882	Hydrogen bond
TLR4:TRP351:NE1	RT:GLN428:OE1	RTB	2.27258	Hydrogen bond
TLR4:ASN356:ND2	RT:ASP500:OD2	RTB	2.42735	Hydrogen bond
TLR4:THR357:N	RT:SER526:OG	RTB	2.84165	Hydrogen bond
TLR4:THR357:OG1	RT:ASN524:OD1	RTB	2.33051	Hydrogen bond
TLR4:ASN427:ND2	RT:ASP377:OD1	RTB	3.00993	Hydrogen bond
TLR4:TYR439:OH	RT:ASP83:OD2	RTA	2.43764	Hydrogen bond
TLR4:ARG512:NE	RT:GLU134:OE1	RTA	2.71612	Hydrogen bond
TLR4:ARG512:NH1	RT:GLY110:O	RTA	2.90917	Hydrogen bond
TLR4:LYS533:NZ	RT:MET40:SD	RTA	2.99009	Hydrogen bond
TLR4:ARG86:CD	RT:SER543:O	RTB	2.85600	Hydrogen bond
TLR4:HIS424:CD2	RT:ASP339:OD2	RTB	2.86625	Hydrogen bond
TLR4:HIS527:CE1	RT:SER355:O	RTB	3.43343	Hydrogen bond
TLR4:TYR449:OH	RT:TRP351	RTB	4.13152	Hydrogen bond
TLR4:SER509:OG	RT:TYR183	RTA	3.55468	Hydrogen bond
TLR4:ILE511:CG2	RT:HIS158	RTA	3.33491	Hydrophobic
TLR4:LEU539:CD2	RT:PHE62	RTA	3.53391	Hydrophobic
TLR4:PRO33	RT:PRO297	RTA	3.93392	Hydrophobic
TLR4:MET40	RT:LEU541	RTB	5.33807	Hydrophobic
TLR4:LYS263	RT:LEU519	RTB	4.69888	Hydrophobic
TLR4:ALA71	RT:PRO27	RTA	4.23869	Hydrophobic
TLR4:ARG74	RT:ILE29	RTA	5.19910	Hydrophobic
TLR4:TRP81	RT:ILE413	RTB	4.82280	Hydrophobic
TLR4:HIS429	RT:PRO352	RTB	5.47002	Hydrophobic
TLR4:TRP407	RT:VAL31	RTA	5.11514	Hydrophobic
TLR4:TYR542	RT:VAL133	RTA	4.98381	Hydrophobic

In previous studies, we have verified the interaction between RT and TLR4 through immunoprecipitation (Dong et al., 2019), which supports the molecular docking model of RT and TLR4 in this section.

## Discussion

RTB is a glycoprotein that delivers RTA to the cytoplasm of host cells *via* glycoproteins and glycolipids located at the cell surface (Olsnes et al., 1975; Hartley and Lord, 2004). Similar to the effects of other bacterial and plant AB toxins on the immune response, we found that recombinant RTB possesses several immunostimulatory functions (Liu et al., 2013; Xu et al., 2013). In this study, we identified a novel TLR4 activator, RTB. We showed that recombinant RTB induced TNF-α production and NF-κB activation in a TLR4-dependent manner in RAW264.7 macrophages.

TLR4 is commonly expressed on macrophages, dendritic cells and other cells. TLR4 signaling pathways are known to play important roles in immune cell activation (Kawai and Akira, 2010; Gay et al., 2014). In previous studies, we have verified the interaction between RT and TLR4 through immunoprecipitation (Dong et al., 2019). In this study, when RAW264.7 cells were treated with RTB, it was observed that recombinant RTB stimulated expression of the TLR4 protein. We further studied the immunomodulatory effect of RTB on TLR4 signaling pathway to clarify its mechanism. First, the Alamar Blue analysis method was used to identify that RTB was not toxic to cells. The expression of TLR4 induced by RTB was enhanced by western blot. The maximum expression of TLR4 was observed at 3 h and gradually decreased until 6 h. To determine whether RTB activates the TLR4 signaling pathway, we examined whether downstream signaling molecules MyD88, IRAK, and TRAF6 were stimulated by RTB. RTB-treated macrophages showed increases in the expression of MyD88, IRAK, and TRAF6. MyD88, IRAK, and TRAF6 protein expression all showed a similar result to TLR4, reaching their maximum level at 3 h and then decreasing gradually up to 6 h. These data indicated that TLR4 might mediate the biological effects of RTB on macrophages. TNF-α is produced in response to LPS through a TLR4/MyD88-dependent pathway (Park et al., 2010). We found that RTB-induced TNF-α secretion was inhibited by treating macrophages with the MyD88 inhibitor Pepinh-MYD and TAK-242, and inhibition of TNF-α secretion was observed in TLR4-/- mouse macrophages. Furthermore, the docking of RT and TLR4 was simulated by computer and the contact residues were concentrated on RTB. The simulation results found that the surface contact residues of TLR4 and RT were concentrated on the B subunit. These data suggest that RTB stimulates TNF-α production by RAW264.7 cells *via* the TLR4 signaling pathway and that TLR4 might act as an RTB receptor.

TLR4 recruits MyD88 through the TIR domain. MyD88 then activates IRAK and TRAF6, which activates MAPK and various transcription factors such as NF-κB (Putra et al., 2014; Yanagibashi et al., 2015; Sakai et al., 2017). IκB-α is an inhibitory protein bound to dimers of NF-κB that retains NF-κB dimers in the cytoplasm. The NF-κB pathway is activated by increasing the level of IκB-α phosphorylation, which causes the release of NF-κB and the expression of cytokine genes, such as TNF-α, IL-1, and IL-6 (Yamamoto and Gaynor, 2004; Kuan et al., 2012; Karin and Delhase, 2012). Our previous studies reported that NF-κB pathways may be involved in RTB-induced TNF-α production. In accordance with our previous study, IκB phosphorylation was altered in RTB-treated macrophages; this effect could be reversed in TLR4- siRNA treated macrophages after RTB treatment. These data suggest that NF-κB is an important signaling pathway in RTB-treated RAW264.7 cells.

The present study demonstrates the mechanism of RTB and TLR4, the induction of TNF-α production by RTB, and the correlation between TNF-α production and activation of the TLR4-dependent pathway induced by RTB stimulation. RTB stimulated macrophages through TLR4-dependent NF-κB activation. These signaling pathways may be responsible for TNF-α production. These data suggest that RTB activates macrophages to trigger TNF-α production through interaction with TLR4-mediated pathways.

## Data Availability Statement

The datasets generated for this study are available on request to the corresponding authors.

## Author Contributions

Conceptualization: NX and WL. Methodology: MD. Software: HY. Validation: YW and MD. Resources: YW. Data curation: YW. Writing (original draft preparation): NX and HY. Writing (review and editing): WL. Project administration: NX. Funding acquisition: NX. All authors contributed to the article and approved the submitted version.

## Funding

This work was supported by the National Science and Technology Major Project(no.2018ZX10101003-005), National Natural Sciences Foundation of China (no. 81773630), and Project Agreement for Science & Technology Development, Jilin Province (no. 20180201004 YY).

## Conflict of Interest

The authors declare that the research was conducted in the absence of any commercial or financial relationships that could be construed as a potential conflict of interest.

## Abbreviations

RTB, Ricin toxin binding subunit B; RIP, Ribosome-inactivating protein; RTA, Ricin toxin binding subunit A; iNOS, Inducible NOS; TNF, Tumor necrosis factor; IL, Interleukin; DCs, Dendritic cells; PRRs, Pattern-recognition receptors; TLRs, Toll-like receptors; MyD88, Primary response protein 88; TRIF, TIR-domain-containing adaptor inducing interferon-β; IRAKs, IL-1R associated kinases; TRAF6, Tumor-necrosis factor receptor associated factor;6 FCS, Fetal calf serum; PVDF, Polyvinylidene difluoride.
